# Presence and toxicity of drugs used to treat SARS-CoV-2 in Llobregat River, Catalonia, Spain

**DOI:** 10.1007/s11356-023-25512-9

**Published:** 2023-02-13

**Authors:** Pol Domínguez-García, Reinerio Rolando Rodríguez, Carlos Barata, Cristian Gómez-Canela

**Affiliations:** 1 Department of Analytical and Applied Chemistry, School of Engineering, Institut Químic de Sarrià-Universitat Ramon Llull, Via Augusta 390, 08017 Barcelona, Spain; 2 Institute for Environmental Assessment and Water Research (IDAEA-CSIC), Jordi Girona 18, 08034 Barcelona, Spain

**Keywords:** COVID-19, Pharmaceutical residues, *D. magna*, Risk assessment, Grab sampling, Liquid chromatography coupled to tandem mass spectrometry (LC–MS/MS)

## Abstract

Severe acute respiratory syndrome coronavirus 2 (SARS-CoV-2) is a strain of coronavirus that causes COVID-19 (coronavirus disease 2019), the respiratory illness responsible for the on-going COVID-19 pandemic. In March 2020, it was declared global pandemic, causing millions of deaths. An evident tendency of global pharmaceutical consumption due to COVID-19 pandemic should be seen worldwide, and this increase might suppose an environmental threat. Pharmaceuticals administrated at home or in pharmacies are excreted by faeces and urine after consumption, and wastewater treatment plants (WWTPs) are not able to remove all pharmaceuticals residues that eventually will end up in the aquatic media (rivers and sea). For this reason, analytical techniques such as liquid chromatography coupled to tandem mass spectrometry (LC–MS/MS) have become prominent to identify and quantify pharmaceuticals residues in aquatic matrices. In view of the scarce data on the occurrence of pharmaceuticals used as COVID-19 treatment, the aim of the present study was to evaluate the presence of these class of pharmaceuticals in river water which were dexamethasone, prednisone, ciprofloxacin, levofloxacin, remdesivir, ritonavir, lopinavir, acetaminophen, hydroxychloroquine, chloroquine and cloperastine, their toxicity in the aquatic environment using *D. magna* and to perform an exhaustive risk assessment in seven points of the Llobregat river basin. Dexamethasone, cloperastine and acetaminophen were the pharmaceuticals with higher concentrations, showing mean levels between 313 and 859 ng L^−1^.

**Supplementary Information:**

The online version contains supplementary material available at 10.1007/s11356-023-25512-9.

## Introduction

Severe acute respiratory syndrome coronavirus 2 (SARS‑CoV‑2) (Gorbalenya et al. 2020) is a strain of coronavirus that causes COVID-19 (coronavirus disease 2019), the respiratory illness responsible for the on-going COVID-19 pandemic. The virus previously had a provisional name, 2019 novel coronavirus (2019-nCoV), and has also been called the human coronavirus 2019 (HCoV-19 or hCoV-19). First identified in the city of Wuhan, Hubei, China, the World Health Organization (WHO) declared the outbreak a public health emergency of international concern on January 30, 2020, and a pandemic on March 112020 (WHO 2020). SARS‑CoV‑2 is a positive-sense single-stranded RNA virus that is contagious in humans (Machhi et al. 2020).

There is an estimation of 6.24 million deaths caused by this infection and more than 516 million of total infections (data from May 2022) (ourworldindata 2022)). SARS-CoV-2 cause severe respiratory syndrome and may affect several systems such as cardiovascular, haematological, nervous, gastrointestinal, renal and hepatobiliary systems (Cascella et al. 2022). Also, due to genetic evolution, the development of new mutations over time results in new variants of the virus in a very short period of time (Cascella et al. 2022). This new global threat directly affects the daily life of billions of people. Previous reports have shown a severe impact in the quality of life of thousands of people, increasing the consumption of substances such as alcohol, stimulant drinks, illegal drugs or pharmaceuticals (Fernandes et al. 2021). In the same study, Fernandes et al. reported an increase of the 23.3% in Portuguese population that expressed the necessity to take any type of therapeuftical drug during the pandemic time (Fernandes et al. 2021). In another publication, authors reported an increase in paracetamol (198%) and hydroxychloroquine (387%) consumption, two pharmaceuticals widely used to combat SARS-CoV-2, during the first wave of the pandemic in Athens, Greece (Galani et al. 2021).

An evident tendency of global pharmaceutical consumption due to COVID-19 pandemic occurred worldwide, which might suppose an environmental threat. Pharmaceuticals administrated at home or in pharmacies are excreted by faeces and urine after consumption, and wastewater treatment plants are insufficient to eliminate all pharmaceutical residues that eventually will end up in the aquatic media (rivers and sea) (Gómez-Canela et al. 2020). In addition, for most places worldwide, wastewater treatment is not done, leading to even worse scenario. For this reason, analytical techniques such as liquid chromatography coupled to tandem mass spectrometry (LC–MS/MS) have become prominent to identify and quantify pharmaceutical residues in aquatic matrices. Good selectivity and very low detection limits of this technique are crucial for this kind of analysis because of the low concentration of some pharmaceuticals in river water (Gros et al. 2006; Sousa et al. 2011; Gómez-Canela et al. 2021).

So far, scarce information can be found on the toxicity and stability of these compounds in water. This is important for both analytical and toxicological evaluations because a risk analysis cannot be performed for this emerging family of contaminants. *Daphnia magna*, which is a planktonic crustacean (Ebert 2005), is one of the most widely used toxicological models in aquatic toxicology due to its reliability and sensitivity (OECD 2004). Moreover, *D. magna* is easy to manage and have a quick reproduction, it is sensitive, and its behaviour/response to many toxic chemicals is known (Baird and Barata 1998). *D. magna* can be used to evaluate the lethal concentration effects (LC_50_) for single and complex mixtures using predictive modeling approaches (Cristale et al. 2013a).

In view of the scarce data on the occurrence of pharmaceuticals used as COVID-19 treatment, the aim of the present study was to evaluate the presence of these new classes of pharmaceuticals in river water, their toxicity in the aquatic environment using the organism *D. magna* and to perform an exhaustive risk assessment in the main points of the Llobregat river (one the main rivers in Catalonia, Spain). To the best of the author’s knowledge, this is the first time that the presence of pharmaceuticals used in the treatment of COVID-19 has been evaluated in a river from Catalonia.

## Experimental

### Chemicals and materials

The 11 pharmaceuticals studied are classified following their Anatomical Therapeutic Chemical Classification code (ATC). They all were purchased from Sigma-Aldrich (St. Louis, MO, USA) with a purity range of 98–99%. Furthermore, atenolol-d7, lidocaine-diethyl-d10 and acetaminophen-methyl-d_3_ were acquired also at Sigma-Aldrich and were used as internal standards. HPLC grade methanol and acetonitrile (ACN) were supplied by VWR Chemicals (Leuven, Belgium). Ammonium formate (NH_4_COOH) and ammonium hydroxide (NH_4_OH) were supplied by Sigma-Aldrich (St. Louis, MO, USA). Hydrochloric acid 37% (HCl) and formic acid (HCOOH) were purchased from Fisher Scientific (Bridgewater, MA, USA). Ultra-pure Milli-Q water was obtained through a Millipore purification system (Millipore, Bedford, MA, USA). Standard stock solutions were prepared in amber glass vials at a concentration of 1000 mg L^−1^ in methanol, and working solutions were prepared between 1 and 2500 µg L^−1^ in 90% of Milli-Q water and 10% of methanol.

### Sampling, pre-treatment and extraction of river samples

Surface water samples were collected in Llobregat River, the second longest river in Catalonia, Spain. Llobregat River was selected because is the most important drinking water source for Barcelona province and, therefore, it flows through different areas of high density of population and industrial zones. It flows into the Mediterranean south of the city of Barcelona. Seven points were sampled along the river length (170 km) starting at Sallent (Barcelona, Spain) and finishing in the Llobregat mouth (A–G). Sampling was repeated three times during the same period of time from November 2021 to February 2022 (1–3). Surface water was sampled from shore stream following the grab sampling methodology and stored in 1-L amber bottles and kept refrigerated until further extraction to avoid the possible degradation of target compounds.

On the other hand, river water samples were filtered using 0.45-µm nylon filters. Each river sample was adjusted at pH 2 with HCl 37% and at pH 7, both using a pH meter SensionTM + PH3(HACH®, Colorado, CO, USA). The extraction method followed a previous published paper focused on the characterization of 76 pharmaceuticals and metabolites in wastewater, with little modifications (Gómez-Canela et al. 2021) (see “Experimental” – “*Extraction procedure*” in Supplementary Information, SI). In order to validate the extraction method, 50 mL or river water samples were spiked at 4 µg L^−1^ with a mix of all target compounds and the mix of internal standards at 0.2 µg L^−1^.

### Analytical performance

Target pharmaceuticals were measured using liquid chromatography with a triple quadruple mass spectrometer detector (Xevo TQS, Acquity H-Class, Waters, Milford, CT, USA) (LC–MS/MS). For the chromatographic separation, a CORTECS T3 column was used (100 mm × 2.1 mm, particle size 1.6 µm, Waters, Milford, CT, USA). The mobile phase consisted of binary mixtures of water with 0.1% HCOOH (A) and acetonitrile with 0.1% HCOOH (B). Gradient elution started at 95% A and 5% B, increasing to 50% B in 7 min, held to 50% B until 12 min and to 100% of B in 3 min and returned to initial conditions in 2 min, with a holding time of 5 min. Figure SI1 displays the chromatographic gradient used for the separation of pharmaceuticals. Flow rate of 300 µL min^−1^ was used, and 10 µL was injected. All the compounds were measured under positive electrospray ionization (ESI +). Cone voltage (C.V.) was optimized from 1 to 90 V to obtain the precursor ion for each target compound under flow injection analysis (FIA). Moreover, the collision energy (C.E.) was optimized from 1 to 40 eV in order to obtain the two most intense fragment ions. Following the acquisition by selected reaction monitoring (SRM), two transitions from the precursor ion to the product ion were used to identify each target compound. The optimal parameters are displayed in Table SI1 for the 11 pharmaceuticals and internal standards. On the other hand, Table SI1 shows the mass fragmentation of the target compounds. The desolvation temperature was set at 350 °C whereas the desolvation gas flow and the cone gas flow were optimized at 900 L h^−1^ and 150 L h^−1^, respectively. Data was processed using MassLynx v4.1 software package.

### Quality assurance

Calibration was performed over a concentration range from 1 to 2500 µg L^−1^ using ten calibration points in MeOH/Milli-Q® water 10:90 (v/v) except from chloroquine and hydroxychloroquine which the range was from 50 to 2500 µg L^−1^ using eight calibration points. Recoveries of pharmaceuticals were estimated using Llobregat river water samples spiked at 4 µg L^−1^ with the mixture of pharmaceuticals. Three internal standards (acetaminophen-(methyl-d_3_), atenolol-d_7_ and lidocaine-d_10_) were used as extraction and analytical control at 0.2 µg L^−1^, and finally, external calibration was used for the pharmaceutical quantification. The instrumental detection limit (IDL) is the minimum amount of analyte required to produce a signal distinguishable from the background noise level within a specified confidence level (Belter et al. 2014). IDL was determined using the lowest concentration of a standard solution that generated an S/N ratio equal to 3 (1 µg L^−1^ except for chloroquine and hydroxychloroquine where the lowest point was 50 µg L^−1^). On the other hand, method detection limit (MDL) was calculated from the injection of spiked river water samples at 4 µg L^−1^ using the minimum concentration of analyte providing an S/N ratio of 3 for the MDL and an S/N ratio of 10 for the limit of quantification (LOQ). The precision of the method was determined by intra-day test, expressed as the percentage relative standard deviation (%RSD) of three replicate injections. The variation was assessed by three consecutive injections of 1 mg L^−1^ standard solution. Finally, matrix effect (ME) was calculated in order to evaluate the degree of signal suppression or enhancement. The ME was calculated by dividing the areas of each pharmaceutical in a solution in river water following Eq. (1). Values close to 100% indicate that there is no matrix effect. However, values higher than 100% means ion enhancement whereas values lower than 100% indicate ion suppression:

1 $$ME \;\left(\%\right)=\frac{A-B}{C} \times 100$$

*A* is the peak area of each analyte from spiked river water samples, *B* is the peak area of each analyte from non-spiked river water, and *C* is the peak area of each analyte in the standard solution. Table 2 displays the quality parameters for each studied pharmaceutical.

### Toxicological studies

Toxicity tests using *D. magna* were performed for dexamethasone, prednisone, ciprofloxacin, levofloxacin, lopinavir, acetaminophen, hydroxychloroquine, chloroquine and cloperastine, which were the most ubiquitous contaminants in the studied Llobregat River. We followed the *Daphnia* sp. Acute Immobilisation Test (OECD 202), which used immobilization as an endpoint, and it is not subject to animal ethical constrains, thus compiling with the 3Rs rules. Two independent sets of experiments were performed, which included the toxicity study for single substances and for mixtures. For single substances, standardized 48-h acute assays were used where neonates < 24 h old were exposed to freshly prepared solutions, and their survival was monitored at 48 h. Single-compound dose-responses were then fitted to the Hill regression model (Eq. 2) to obtain accurate concentration dose–response curves.

In a second experiment, multicomponent mixtures of the nine studied compounds were assayed using the ray design, in which exposure levels were selected to include constant equitoxic (EC_50_) mixture ratios and 8 different mixture effect levels, which allow consideration of explicit concentration–response relationships. This design is best suited to comparing responses with the concentration addition (CA) and independent action (IA) concepts. Both concepts predict non-interactive joint additive effects of similar (CA) and dissimilar (IA) acting chemicals and are widely used in aquatic toxicology (Altenburger et al. 2003).

All dilutions are reported as nominal concentrations. Stock solutions (2000 ×) of the individual chemicals or mixtures were prepared in water on the day of the experiment.

The concentration–response relationships of the individual substances were biometrically modelled by using a best-fit approach (Scholze et al. 2001) and the Hill model of Eq. 2:

2 $$E\left(\%inh\right)=\frac{100}{1+{\left({EC}_{50}/x\right)}^{p}}$$

with *E* = effect in %; *p* = slope; *EC* = lethal effect concentration; *x* = concentration (µM).

On the basis of the concentration–response functions of individual compounds, predictions of concentration addition were calculated for mixture containing binary combinations in a definite ratio (based on EC_50_). A total concentration of the mixture, at which a certain effect is generated, can be calculated using CA according to Eq. 3:

3 $${ECx}_{mix}={\left({\sum }_{i=1}^{n}\frac{{p}_{i}}{{EC}_{xi}}\right)}^{-1}$$

In this equation, *ECx*_*mix*_ is the total concentration of the mixture provoking *x*% lethal effect; *ECxi* is the concentration of component *i* provoking the *x*% lethal effect, when applied singly; and *p*_*i*_ denotes the fraction of component *i* in the mixture. The calculation of total mixture concentrations for various lethal effect levels leads to a complete iteration of an expected concentration–lethal effect relationship.

The prediction concept IA allows explicit calculation of combined effects according to Eq. 4:

4 $$E({c}_{mix})=1-\prod_{i=1}^{n}\left(1-E({c}_{i})\right)$$

The lethal effect at the total concentration of the mixture, *E*(*c*_*mix*_), is based on the lethal effects of the components which they generate at concentration *x* at which they are present in the mixture (*E*(*ci*)). If the latter is expressed as a fraction (*pi*) of the total mixture concentration, it holds Eq. 5:

5 $$L({c}_{mix})=1-\prod_{i=1}^{n}\left(1-E({p}_{i}{c}_{mix})\right)$$

This allows calculation of a lethal effect expected according to the concept of response addition for any concentration of the mixture.

### Risk assessment

Risk assessment was calculated in order to evaluate the potential risk of the target pharmaceuticals in the environment. Risk assessment is approached via the calculation of the risk quotient (*RQ*) based on the measured environmental concentration (MEC) and the predicted no effect concentration (PNEC) (Thomaidi et al. 2017). PNEC can be estimated as the toxicological relevant concentration (EC_50_) and a security factor (*f* = 1000) used for compensation of the few chronic toxicity as PNEC values refer to acute toxicity of the organisms (Nika et al. 2020). Equation 6 shows the *RQ* formula:

6 $$RQ=\frac{MEC}{PNEC}=\frac{MEC}{{EC}_{50/f}}$$

Results were interpreted following the maximum probable risk for ecotoxicological effects from contaminated water (Marcus et al. 2010). This result is the ratio of the potential exposure to the pharmaceutical and the level at which no adverse effects are expected, where *RQ* < 1 indicates no significant risk, values between 1 ≤ *RQ* < 10 indicate a small potential for adverse effects, values between 10 ≤ *RQ* < 100 indicate potential for adverse effects and finally, *RQ* ≥ 100 indicates the potential for adverse effects.

## Results and discussion

### Quality parameters

Table 1 shows the different quality parameters for the 10 pharmaceuticals studied. The linearity was in the range of 1 to 2500 µg L^−1^ with good correlation (*R*^2^ ≥ 0.99) for dexamethasone, prednisone, ciprofloxacin, levofloxacin, remdesivir, ritonavir, lopinavir, acetaminophen, and cloperastine. However, linearity for hydroxychloroquine and chloroquine ranged between 50 and 2500 µg L^−1^ with also a good correlation (*R*^2^ ≥ 0.98). IDL value ranged between 4.57 pg (remdesivir) and 21.2 pg (cloperastine). On the other hand, MDL values were between 2 ng L^−1^ (chloroquine) and 24 ng L^−1^ (dexamethasone) and LOQ values between 8 and 80 ng L^−1^ (chloroquine and dexamethasone, respectively). Furthermore, intra-day precision of the chromatographic method was ranged between 1 and 11% (*N* = 3). Otherwise, matrix effect values were between 31% (levofloxacin) and 106% (ritonavir), indicating signal suppression for some pharmaceuticals. Finally, recoveries were between 33% (chloroquine) to 91% (ritonavir) at pH 7 which had the higher recovery rates (Table 1). For a method with 10 pharmaceuticals as they are usually classified for their mechanism of action and can have very different physicochemical properties among them, it is considered that recoveries over 30% are acceptable. Blank did not show any signal for any of pharmaceuticals.

**Table 1:** Quality parameters obtained for 11 pharmaceuticals ordered following de ATC code. *DXM* dexamethasone, *PRED* prednisone, *LEV* levofloxacin, *CIP* ciprofloxacin, *RDV* remdesivir, *RTV* ritonavir, *LPV* lopinavir, *APAP* acetaminophen, *HCQ* hydroxychloroquine, *CHQ* chloroquine, *CPS* cloperastine

Compound	Linearity (µg L^−1^)	*R*^2^	IDL (pg)	Intra-day precision (%)	%R ± RSD	MDL (ng L^−1^)	LOQ (ng L^−1^)	Matrix effect (%)
Dexamethasone	0.001–2.5	0.9989	20.8	7	52 ± 8	24	80	41
Prednisone	0.001–2.5	0.9994	6.82	1	65 ± 2	14	47	60
Ciprofloxacin	0.001–2.5	0.9899	12.3	6	89 ± 7	7	59	62
Levofloxacin	0.001–2.5	0.9933	10.0	9	40 ± 2	5	16	31
Remdesivir	0.001–2.5	0.9996	4.57	8	45 ± 3	6	20	46
Ritonavir	0.001–2.5	0.9970	16.4	1	91 ± 2	7	24	106
Lopinavir	0.001–2.5	0.9998	5.82	6	58 ± 1	1	4	60
Acetaminophen	0.001–2.5	0.9934	12.8	5	71 ± 7	12	39	56
Hydroxychloroquine	0.05–2.5	0.9750	5.33	1	78 ± 4	4	12	64
Chloroquine	0.05–2.5	0.9948	5.38	1	33 ± 11	2	8	37
Cloperastine	0.001–2.5	0.9980	21.2	11	77 ± 5	3	11	55

### Presence of pharmaceuticals in Llobregat River

In this study, 11 pharmaceuticals specifically and non-specifically used for COVID-19 pandemic were monitored in Llobregat river (Barcelona, Catalonia, Spain), which were (ordered by ATC code) dexamethasone, prednisone, ciprofloxacin levofloxacin, remdesivir, ritonavir, lopinavir, acetaminophen, hydroxychloroquine, chloroquine and cloperastine. Table 2 shows the concentrations of the 11 pharmaceuticals in river water. These results must be taken as time independent punctual results using grab sampling mode. Factors such as weather conditions or river flow stream are not considered and that is the reason for the three different samplings on different days.

**Table 2:** Concentrations of pharmaceuticals in river water (in ng L^−1^). Sampling points (A–G). Sampling collection time (1–3). LL (Llobregat River). *DXM* dexamethasone, *PRED* prednisone, *LEV* levofloxacin, *CIP* ciprofloxacin, *RDV* remdesivir, *RTV* ritonavir, *LPV* lopinavir, *APAP* acetaminophen, *HCQ* hydroxychloroquine, *CHQ* chloroquine, *CPS* cloperastine

Compound (ng L^−1^)	DXM	PRED	CIP	LEV	RDV	RTV	LPV	APAP	HCQ	CHQ	CPS
LL_1_A	< LOQ	117.0	< LOQ	48.77	< LOQ	< LOQ	< LOQ	543.9	< LOQ	16.81	196.5
LL_1_B	< LOQ	< LOQ	< LOQ	11.12	< LOQ	< LOQ	< LOQ	98.88	14.62	< LOQ	268.4
LL_1_C	< LOQ	91.87	< LOQ	12.65	< LOQ	< LOQ	< LOQ	484.9	< LOQ	39.25	264.9
LL_1_D	< LOQ	< LOQ	< LOQ	97.38	< LOQ	< LOQ	< LOQ	459.3	< LOQ	17.08	841.2
LL_1_E	< LOQ	96.31	< LOQ	19.02	< LOQ	< LOQ	< LOQ	520.3	< LOQ	10.58	603.7
LL_1_F	< LOQ	186.6	96.02	38.75	< LOQ	< LOQ	< LOQ	140.5	< LOQ	1015	504.5
LL_1_G	< LOQ	133.8	< LOQ	11.86	< LOQ	< LOQ	< LOQ	580.8	< LOQ	< LOQ	1296
LL_2_A	189.1	< LOQ	< LOQ	7.795	< LOQ	< LOQ	< LOQ	766.3	< LOQ	< LOQ	< LOQ
LL_2_B	155.0	< LOQ	< LOQ	86.38	28.46	< LOQ	16.47	502.5	95.85	43.90	85.08
LL_2_C	101.6	< LOQ	< LOQ	44.61	< LOQ	< LOQ	7.316	482.8	< LOQ	< LOQ	80.59
LL_2_D	66.94	< LOQ	< LOQ	92.34	< LOQ	< LOQ	< LOQ	642.2	< LOQ	< LOQ	< LOQ
LL_2_E	115.7	< LOQ	< LOQ	31.50	< LOQ	< LOQ	< LOQ	192.9	12.84	< LOQ	181.3
LL_2_F	362.9	< LOQ	87.00	56.91	< LOQ	< LOQ	< LOQ	3377	< LOQ	< LOQ	< LOQ
LL_2_G	60.33	< LOQ	< LOQ	8.356	< LOQ	< LOQ	< LOQ	91.85	< LOQ	< LOQ	< LOQ
LL_3_A	168.4	< LOQ	< LOQ	30.48	88.87	91.74	92.38	< LOQ	29.78	96.57	101
LL_3_B	550.2	135.3	< LOQ	55.33	< LOQ	< LOQ	5.532	2085	12.54	< LOQ	< LOQ
LL_3_C	482.1	< LOQ	< LOQ	38.22	< LOQ	< LOQ	10.90	647.2	< LOQ	< LOQ	< LOQ
LL_3_D	530.1	< LOQ	< LOQ	63.39	< LOQ	< LOQ	13.69	1232	< LOQ	78.49	97.09
LL_3_E	477.5	267.6	< LOQ	82.83	< LOQ	< LOQ	13.10	645.5	< LOQ	62.56	107.3
LL_3_F	657.8	< LOQ	< LOQ	118.0	< LOQ	< LOQ	47.44	494.0	< LOQ	< LOQ	241.0
LL_3_G	469.1	< LOQ	< LOQ	54.49	< LOQ	< LOQ	33.91	3198	< LOQ	< LOQ	530.9
Mean	313.4	146.8	92.51	48.10	58.66	91.74	26.75	859.3	33.12	153.3	359.9
*s*	227.2	78.50	27.56	32.49	20.06	20.02	22.37	933.3	21.57	219.38	332.8
RSD (%)	72.50	53.48	30	67.55	34.20	21.82	83.63	108.6	65.11	143.1	92.47

Acetaminophen, which is one of the most consumed analgesics around the world, had increased worldwide because of its analgesic properties (Mostafa et al. 2022). Acetaminophen gave the highest concentrations ranging from 91.85 to 3377 ng L^−1^ (LL2G and LL2F respectively), with a mean value of 859.3 ng L^−1^. Only in LL3A, acetaminophen was not detected. In 2017, Al-Kaf et al. reported a maximum value of acetaminophen at 2420 ng L^−1^ in Llobregat river (Al-Kaf et al. 2017).

Following acetaminophen, cloperastine which is a widely used antihistamine drug also postulated for COVID-19 treatment (Turabian 2020) had concentrations between 80.59 to 1296 ng L^−1^ (LL2C and LL1G respectively), with a mean value of 92.47 ng L^−1^.

On the other hand, dexamethasone, which is a long-acting glucocorticoid that is administrated to decrease hyperinflammation and as a immunosuppressive (Mehta et al. 2022), gave values between 60.33 ng L^−1^ (LL2G) and 657.8 ng L^−1^ (LL3F). Desgens-Martin et al. studying the potential environmental risk caused by COVID-19 treatment agents estimated that dexamethasone was expected to have a maximum peak of 55.6 ng L^−1^ (data from January 2021 in surface water) (Desgens-Martin and Keller 2021). Before pandemic, Herrero et al. reported that dexamethasone was not expected to have concentrations higher than 20 ng L^−1^ in a study of glucocorticoids in sewage and river water (Herrero et al. 2012). Mean value of dexamethasone obtained in this study suggests a huge increase of this pharmaceutical in river water (313.4 ng L^−1^), see Table 2.

Following dexamethasone, chloroquine had a range of concentrations in river water between 10.58 and 1015 ng L^−1^ (LL1E and LL1F, respectively), with a mean value of 153.3 ng L^−1^. It was detected in 9 out of 21 samples. In a previous study about the determination of pharmaceuticals in underground water from Nigeria, in 2014, chloroquine was detected at concentrations of 110 ng L^−1^ (Olaitan et al. 2014). The predicted environmental concentration (PEC) of chloroquine reports an estimation of 32 ng L^−1^ in surface water but considering a very effective WWTP removal rate (Kumar et al. 2020).

Prednisone, which is a corticosteroid used for the treatment of a wide range of conditions, including inflammatory conditions, allergic reactions, autoimmune conditions and even certain types of cancer (Erdoğan et al. 2019) gave values between 91.87 and 267.6 g L^−1^ (LL1C and LL3E respectively). However, in 15 out of 21 samples, prednisone had values below the LOQ. Data from around the world about glucocorticoids’ levels in the environment reported by Yazdan et al. ranged the concentrations of prednisone in river water from 0.2 to 100 ng L^−1^ (Yazdan et al. 2021). The mean value of prednisone was 146.8 ng L^−1^ in this study.

On the other hand, ciprofloxacin, a quinoline used for bacterial infections such as urinary tract infections or pneumonia (Thai et al. 2022), was detected in LL1F and LL2G with levels of 96.02 and 87.00 ng L^−1^ (Table 2), respectively. During the pandemic, ciprofloxacin was used for combat SARS-CoV-2 virus with patients with respiratory disease (Mustafa et al. 2021). Kenyon et al. reported that mean concentrations of ciprofloxacin in Spain were lower than 10.1 ng L^−1^ (Kenyon et al. 2022). In this study, we obtained a mean value of 92.51 ng L^−1^ which indicated an increase of this pharmaceutical regarding to previous studies.

Otherwise, ritonavir, a protease inhibitor widely used for HIV infection, was also administrated during the pandemic for the treatment of COVID (Patel et al. 2021). Ritonavir only was detected in one sample with a value of 91.74 ng L^−1^ in LL3A. In a previous study, Aminot et al. reported a concentration of 0.2 ng L^−1^ for ritonavir in river water from France (July 2012) (Aminot et al. 2015).

Remdesivir is a brad-spectrum antiviral that inhibits the RNA polymerase of a virus. Only in two points remdesivir was detected at higher levels than its LOQ which gave concentrations of 28.46 and 88.87 in LL2B and LL3A, respectively (Table 2). Concentrations of remdesivir were reported from 430 to 2120 ng L^−1^ in surface water during pandemic time (Morales-Paredes et al. 2022). A mean value of 58.66 ng L^−1^ is measured in this study, but it was only detected in two samples from Llobregat River. CatSalut (The Catalan health service) reported unclear therapeutic benefits of this pharmaceutical in patients whose health was severely impaired by SARS-CoV-2 and did not recommend its administration (Catal 2022).

Levofloxacin is a quinolone antibiotic used to treat bacterial infections in many parts of the body like pulmonary infection (IMB Watson Health 2022). Levofloxacin concentrations in river water ranged from 7.795 to 118.0 ng L^−1^ (LL2G and LL3F respectively). In a previous study, Lacorte et al. reported a PEC value from levofloxacin in river water was 14 ng L^−1^ in Spain from a study of pharmaceuticals in wastewaters from senior residences (Lacorte et al. 2018). Hann et al. reported a value of levofloxacin of 6.0 ng L^−1^ in river waters from China (Hanna et al. 2018). Mean value for levofloxacin in this study was 48.10 ng L^−1^ (Table 2) which is higher than those predicted or measured in river water in the previous mentioned two studies.

On the other hand, hydroxychloroquine is an antiprotozoal widely used for malaria and amebiasis treatment (Liu et al. 2020). Its concentrations in the samples ranged from 12.54 to 95.85 ng L^−1^ (LL2E and LL2B, respectively), with a mean value of 33.12 ng L^−1^. It was detected in 5 out of 21 samples which indicates a low presence of this pharmaceutical. In a previous study, Kuroda et al. reported an estimation of 78.23 ng L^−1^ of hydroxychloroquine in surface water during the pandemic peak (March 2021). In the same way, chloroquine is also an antiprotozoal similar to hydroxychloroquine, and both pharmaceuticals increase the endosomal pH, which inhibits fusion between SARS-CoV-2 and the host cell membrane (NIH 2021).

Finally, lopinavir which is also a protease inhibitor like ritonavir that was suggested for COVID treatment usually accompanied with ritonavir (Patel et al. 2021) gave ranged values between 5.532 and 92.38 ng L^−1^ (LL3B and LL3A, respectively). It was detected in 9 out of 21 samples. PEC data reported from Kuroda et al. establishes a predicted concentration of 2.2 ng L^−1^ of lopinavir (Kuroda et al. 2021). In this study, a mean value of 26.75 ng L^−1^ was obtained (Table 2).

Summarizing the obtained results, every pharmaceutical was detected in at least one river point through the three different samplings. RSD values are high in this study regarding the different sampling points that corresponded to river areas with different population densities, industrialization pressures and the presence of nearby hospitals. That was the reason why three samplings were made in different periods of time.

### Toxicological results

**Fig. 1:**
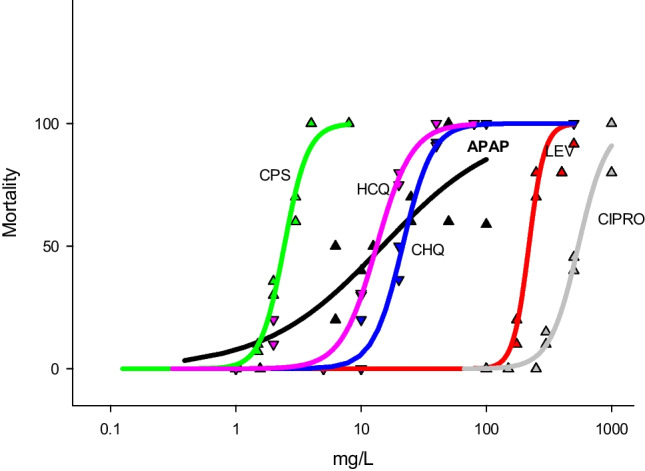
Single-compound *D. magna* toxicity responses of the studied substances: concentration-mortality curves fitted to the Hill regression model are also depicted. Each symbol corresponds to a single value. CPS: cloperastine; HCQ: hydroxychloroquine; CHQ: chloroquine; APAP: acetaminophen; LEV: levofloxacin; CIPRO: ciprofloxacin. The horizontal *X* axis is in log scale

*D. magna* was used as a model organism to evaluate aquatic toxicity. From the 11 target compounds, 9 were detected in most of the samples analysed, and 6 showed toxicity < 1 g L^−1^ which were cloperastine, hydroxychloroquine, chloroquine, acetaminophen, levofloxacin and ciprofloxacin. For individual compounds, mortality responses followed a sigmoid curve (Fig. 1), which could be modelled by the Hill regression function of Eq. (2). In all cases, the residuals of the regression models obtained were normally distributed (Kolmogorov–Smirnov tests *P* > 0.05) giving coefficients of determination higher than 0.8 (Table 3). Toxicity of the 6 tested chemicals differed largely across substances, with EC_50_ values ranging over 2 orders of magnitude between cloperastine (2.4 mg L^−1^) and ciprofloxacin (542 mg L^−1^) (Table 3).

**Table 3:** EC_50_ values (in mg L^−1^), *r*^2^ and sample size for the six compounds whose toxicity was studied

	EC_50_ (mean, mg L^−1^)	SE	*r*^2^	*N*
Acetaminophen	14.8	3.6	0.81	14
Levofloxacin	218	8.9	0.94	12
Cloperastine	2.43	0.98	0.99	16
Chloroquine	21.4	0.79	0.98	16
Hydroxychloroquine	13.3	0.65	0.97	16
Ciprofloxacin	542	19	0.97	16

Until present, toxicity data on the aquatic organisms are available for chloroquine, hydroxychloroquine, levofloxacin, ciprofloxacin and acetaminophen. Zurita et al. reported NOAEL (non-observed adverse effect levels) of chloroquine to several aquatic organisms being *Daphnia magna* the most sensitive organism (2,5 µM ≅ 1 mg L^−1^) (Zurita et al. 2005). Rendal et al. reported a strong effect of pH on toxicity of chloroquine using *D. magna*, where the EC_50_ fell from approximately 30 mg L^−1^ at pH 7 to 4 mg L^−1^ at pH 9 (Rendal et al. 2011). Reported toxic results of hydroxychloroquine are limed to biochemical responses of tadpoles and marine nematodes indicating adverse effects at 10–30 mg L^−1^ (Ben Ali et al. 2021; da Luz et al. 2021). Acute toxicity of ciprofloxacin in *D. magna* has been reported on the range of 30–70 mg L^−1^ (Martins et al. 2012; Dionísio et al. 2020). Both studies also showed that sublethal effects of ciprofloxacin on *Daphnia* reproduction and oxidative stress–related biomarkers occurred at concentrations ten times lower. Levofloxacin was reported to impair *D. magna* reproduction at 340 mg L^−1^ and algae growth at 1 mg L^−1^ (Yamashita et al. 2006). Reported LC_50_ of paracetamol in *D. magna* varied from 10 to 50 mg L^−1^, which are in line with our results. Interestingly, at 2 mg L^−1^, paracetamol also impaired *D. magna* reproduction (Nunes et al. 2014). The above-mentioned reported toxicity results are in line with our toxicity data and indicated that for most compounds, detrimental sublethal can also occur at lower concentrations than those affecting *D. magna* immobility.

**Fig. 2:**
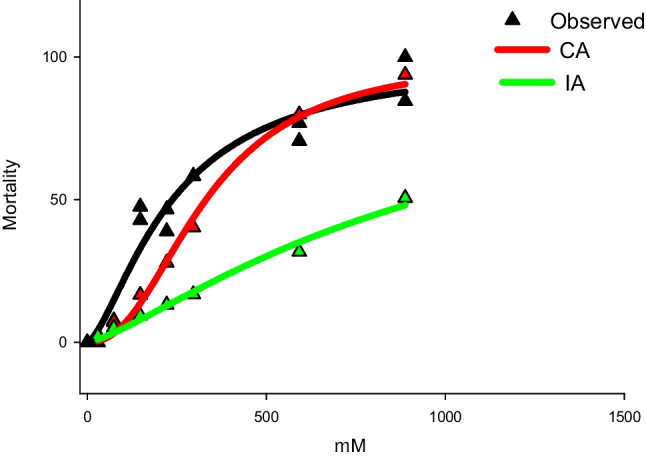
Joint toxicity of the 6 multicomponent mixture. Each point represents a single value. Black, red and green lines are, respectively, observed responses and predicted ones following the concentration addition (CA) and independent action (IA)

Multi-component test mixture responses containing the six studied compounds at the molar ratio of their individual EC_50_ values are depicted in Fig. 2. For convenience, multi-component concentrations are depicted in μM. Observed joint effects for the mixture were closer to joint effect predictions following the CA concept than that of IA. This indicates that the studied pharmaceuticals were toxic to *D. magna* probably throughout similar mechanisms of action (Cristale et al. 2013b). This means that it is correct to estimate the risk assessment of these compounds just summing up their individual risks (Cristale et al. 2013b).

### Risk assessment

A value for the *RQ *has been calculated for each sampling point using the concentrations of pharmaceuticals and the EC_50_ calculated in the previous section (“Toxicological results”). These values can be seen at Table SI2.

Table 3 shows the mean values of the summatory of all the pharmaceuticals in each Llobregat point (A–G). As can be seen in Table 3, all values are lower than 1 which is considered the threshold value for an ecotoxicological risk (Marcus et al. 2010). However, the obtained results have to be taken into consideration as they are not negligible and are based on acute responses. These mean that sublethal toxic effects (i.e. effects on growth and reproduction) are likely to occur at lower concentrations (Roex et al. 2000). Sample locations B, D, E and F had *RQ* higher than 0.1 and sample location G had the highest *RQ* (0.51). These results indicate that they are not an environmental threat at this time, but they should be considered in the future because they may become at risk. Furthermore, from the compounds analysed, the APAP and CPS contributed the most (> 97%) to the total hazard, which means that remediation measures should be taken for these two compounds.

**Fig. 3:**
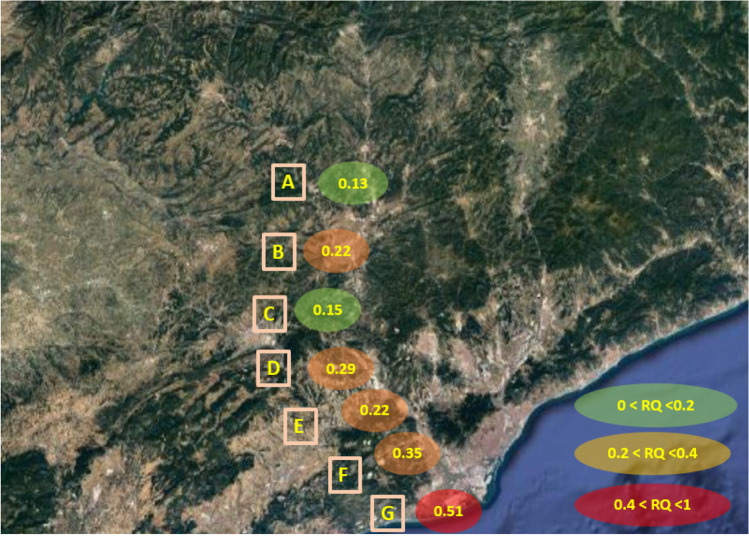
Satellite map of the sampling points and their Σ*RQ* from low to high environmental risk (green to red respectively)

Figure 3 shows the different sampling points on a satellite map and their associated *RQ*. As can be seen at the figure, sampling points near the river mouth increase their *RQ*. Sampling points A to C correspond to areas with lower density of population whereas sampling locations D to F correspond to areas with high demography and had higher *RQ*. Also, point F is picked near a hospital, and *RQ* value increased significantly (0.35). Finally, point G which corresponds to the Llobregat river mouth had the highest *RQ* with a value of 0.51. This means that chemical risks increased towards the river mouth as the number of WWTP discharges increased, and recalcitrant chemical residues not eliminated in wastewater treatment plants and hence present in their effluents are continuously discharged into the river. There is also the fact that in the Llobregat River, heavy populated urban areas (i.e. Barcelona city) are located close to the river mouth. This is common in heavily exploited Mediterranean rivers (IMPREX 2020). Seasonal reductions in water river flow may also increase the contaminant concentration of pharmaceuticals and hence their potential toxicity and risks since their consumption varied little across the year (Quincey et al. 2022). Our study was performed in late autumn and winter, which are characterized by being moderately dry, and thus, the Llobregat river water flow is moderate. Accordingly, we can assure that our risk values may be considered as average yearly estimates (Table 4).

**Table 4:** Summatory of the *RQ* of all pharmaceuticals in each river sampling point

Sampling locations	∑*RQ* mean	*s*	RSD (%)
LLA	0.13	0.07	53
LLB	0.22	0.13	59
LLC	0.15	0.05	33
LLD	0.29	0.19	65
LLE	0.22	0.14	64
LLF	0.35	0.19	55
LLG	0.51	0.42	82

*RQ* risk quotient, *s* standard deviation, *RSD* relative standard deviation

## Concluding remarks

The most widely used pharmaceuticals for COVID-19 treatment during the pandemic have been analysed in Llobregat river. Mean values of all of them were reported in the range of 92–859 ng L^−1^ and the pharmaceutical with highest concentration was acetaminophen, one of the world’s most consumed analgesic. However, ritonavir and remdesivir gave trace concentrations near their quantification limits. No matter what, it has been established that there has been an increase of pharmaceutical residues in surface water due to COVID-19 pandemic as they were detected in much lower concentrations before 2019 in river water. This fact could become a new environmental threat, and most of them should be monotonized in the future because the pandemic is far from over.

Regarding toxicological studies, 6 pharmaceuticals showed toxicity < 1 g L^−1^ which were acetaminophen, levofloxacin, cloperastine, chloroquine, hydroxychloroquine and ciprofloxacin. From all pharmaceuticals, acetaminophen and ciprofloxacin contributed the most (> 97%) to the total hazard, which means that remediation measures should be taken for these two compounds.

## Supplementary Information

Below is the link to the electronic supplementary material.

Supplementary file1 (DOCX 458 KB)

## Data Availability

Data will be made available on request.
