# Supplementary material for: Presence and toxicity of drugs used to treat SARS-CoV-2 in Llobregat River, Catalonia, Spain
Source: Environ Sci Pollut Res Int. 2023 Feb 13;30(17):49487–97. doi: 10.1007/s11356-023-25512-9 (PMC9924204; doi:10.1007/s11356-023-25512-9)
Supplement: Supplementary file 1 — Supplementary file1 (DOCX 458 KB) [file 11356_2023_25512_MOESM1_ESM.docx]

**Supplementary Information (SI)**

**Presence and toxicity of drugs used to treat SARS-CoV-2 in Llobregat River, Catalonia, Spain.**

Pol Dominguez-García^a^, Reinerio Rolando Rodríguez^a^, Carlos Barata^b^, Cristian Gómez-Canela^a,^*

^a^*Department of Analytical and Applied Chemistry, School of Engineering, Institut Químic de Sarrià-Universitat Ramon Llull, Via Augusta 390, 08017 Barcelona, Spain.*

*^b^Institute for Environmental Assessment and Water Research (IDAEA-CSIC), Jordi Girona 18, 08034 Barcelona, Spain.*

*Corresponding author:

E-mail address: cristian.gomez@iqs.url.edu (C. Gómez-Canela)

Tel: +34 93 261 23 43

**EXPERIMENTAL SECTION**

***Extraction procedure***

The extraction consisted in a solid-phase extraction (SPE) with Oasis HLB cartridges (200 mg, 6 cc, Waters, Mildford, MA, USA) in order to preconcentrate target analytes. All cartridges were conditioned with 6 mL MeOH and with 6 mL of Milli-Q^®^ water, as equilibration step. The sample was loaded at flow 1 mL min ^-1^ and then, the cartridges were dried at room temperature for 20 min. The elution was done with 4 mL MeOH, 4 mL MeOH + 0.1% formic acid and 4 mL MeOH + 0.1% NH_4_OH in order to elute the higher number of neutral, basic and acid drugs. Extracts were evaporated under a current of N_2_ to near dryness, re-suspended in 1 mL MeOH and transferred to a chromatographical vial. Finally, samples were evaporated again under N_2_ until dryness and then, reconstituted with 200 µL of a 10:90 (v/v) MeOH:Milli-Q® water solution.

***UHPLC-MS/MS***

Mobile phase consisted of binary mixtures of water with 0.1% formic acid (A) and acetonitrile with 0.1% formic acid (B). Gradient elution started at 95% A and 5% B, increasing to 50% B in 7 min, held to 50% B until 12 min and to 100% of B in 3 min and returned to initial conditions in 2 min, with a holding time of 5 min. Figure SI1 displays the chromatographic gradient. Flow rate of 300 µL min^-1^ was used and 10 µL were injected. All the compounds were measured under positive electrospray ionization (ESI+). Cone voltage (C.V.) was optimized from 1 to 90 V to obtain the precursor ion for each target compound using flow injection analysis (FIA). Moreover, the collision energy (C.E.) was optimized from 1 to 40 eV in order to obtain the two most intense fragment ions. Following the acquisition by selected reaction monitoring (SRM), two transitions from the precursor ion to the product ion were used to identify each target compound. The optimal parameters are displayed in Table 1 for the 11 pharmaceuticals, and internal standards studied. Table SI1 shows the mass fragmentation of the target compounds. The desolvation temperature was set at 350 °C whereas the desolvation gas flow and the cone gas flow were optimized at 900 L h^-1^ and 150 L h^-1^, respectively. The system and data management were processed with MassLynx v4.1 software package.

**Figure SI1.** LC-MS/MS gradient conditions.

**Results and discussion**

**Optimization of ionization parameters and chromatographic conditions**

Optimization of the ionization conditions was performed using LC-MS/MS in ESI+ mode. Table SI1 summarizes the precursor ions and the two most intense fragments, as well as the optimum C.E. and C.V. for each target pharmaceutical. C.V. was adjusted from 6 to 96 V to achieve the best response for the protonated molecule. After, the protonated molecule was fragmented to produce product ions with good intensity by changing the C.E which was optimized from 5 to 46 eV. The two most abundant fragments were monitored for each target compound, one for quantification and the second for confirmation. Table SI2 shows the mass spectral characterization of the target compounds. Finally, Figure SI2 displays the chromatographic peaks of a standard mixture at 1000 µg L^-1^.

**Table SI1.** LC-MS/MS optimized parameters for selected pharmaceuticals (ordered by ATC code), and their internal standards (ˆ). C.V.: cone voltage (V); C.E.: collision energy (eV); q1: product ion for quantification; q2: product ion for confirmation.

| **Target Compound** | **Retention Time (Rt)** | **Precursor Ion** | **C.V (V)** | **q1 Transition** | **C.E. (eV)** | **q2 Transition** | **C.E. (eV)** |
| --- | --- | --- | --- | --- | --- | --- | --- |
| Dexamethasone | 8.61 | 393 | 24 | 393 > 147 | 20 | 393 > 121 | 50 |
| Prednisone | 7.58 | 359 | 7 | 359 > 341 | 11 | 359 > 147 | 36 |
| Ciprofloxacin | 4.93 | 332 | 40 | 332 > 314 | 20 | 332 > 245 | 20 |
| Levofloxacin | 4.66 | 362 | 44 | 362 > 318 | 18 | 362 > 261 | 26 |
| Remdesivir | 10.04 | 603 | 17 | 603 > 402 | 13 | 603 > 229 | 21 |
| Ritonavir | 14.28 | 721 | 50 | 721 > 296 | 16 | 721 > 268 | 28 |
| Lopinavir | 14.58 | 629 | 27 | 629 > 447 | 11 | 629 > 183 | 19 |
| Acetaminophen | 2.71 | 152 | 32 | 152 > 110 | 20 | 152 > 93 | 20 |
| Hydroxychloroquine | 3.68 | 336 | 10 | 336 > 247 | 21 | 336 > 158 | 21 |
| Chloroquine | 3.9 | 320 | 40 | 320 > 247 | 11 | 320 > 142 | 17 |
| Cloperastine | 9.74 | 332 | 42 | 332 > 203 | 18 | 332 > 166 | 45 |
| Acetaminophen-(methyl-d3) ^^^ | 2.98 | 155 | 30 | 155 > 110 | 38 | 155 > 93 | 20 |
| Atenolol-d7 ^^^ | 3.01 | 274 | 34 | 274 > 145 | 26 | 274 > 74 | 22 |
| Lidocaine-d10 ^^^ | 4.81 | 245 | 24 | 245 > 96 | 10 | 245 > 64 | 38 |


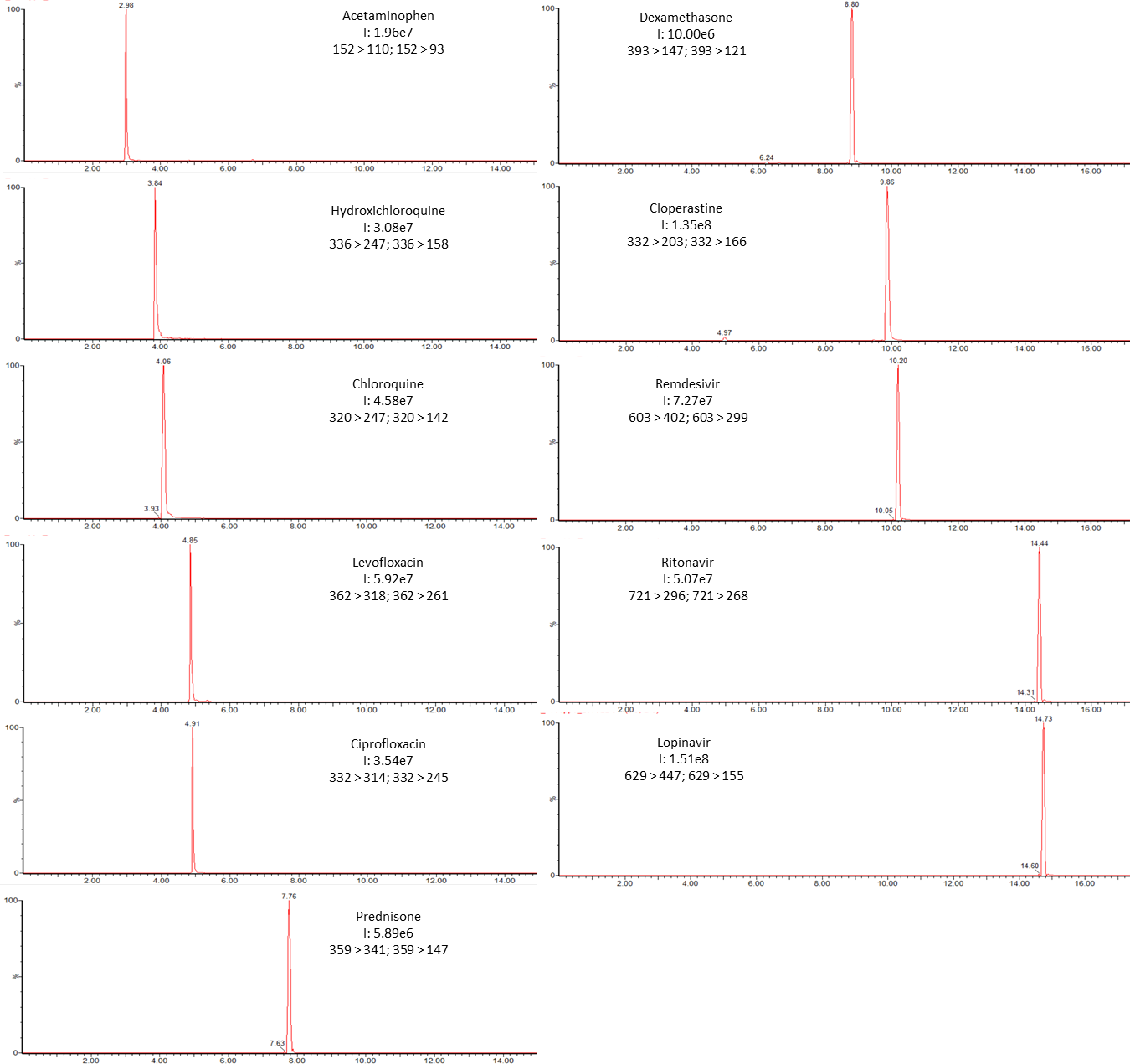


**Figure SI2.** Chromatographic peaks of target compounds with a standard of 1000 µg L^-1^

| **Table SI2**. MS/MS fragmentation of the target compounds. | | |
| --- | --- | --- |
| **Acetaminophen** | **Chloroquine** | **Ciprofloxacin** |
|  |  |  |
| **Cloperastine** | **Dexamethasone** | **Hydroxychloroquine** |
|  |  |  |
| **Levofloxacin** | **Lopinavir** | **Prednisone** |
|  |  |  |
| **Remdesivir** | **Ritonavir** |  |
|  |  |  |
|  |  |  |

**Table SI2.** Values of the RQ in every sampling point and summatory of the values for all pharmaceuticals (Samplings 1-3).

| **Samples** | **Chloroquine** | **Ciprofloxacin** | **Cloperastine** | **Dexamethasone** | **Hydroxychloroquine** | **Levofloxacin** | **Lopinavir** | **Paracetamol** | **Remdesivir** | **Ritonavir** | **Prednisone** | **∑RQ** |
| --- | --- | --- | --- | --- | --- | --- | --- | --- | --- | --- | --- | --- |
| LL1A | 0.00084 | 0 | 0.098 | Non-Toxic | 0.00016 | 0.00028 | Non-Toxic | 0.087 | Non-Toxic | Non-Toxic | Non-Toxic | 0.19 |
| LL1B | 0.0018 | 0 | 0.13 | Non-Toxic | 0.0015 | 0.000064 | Non-Toxic | 0.016 | Non-Toxic | Non-Toxic | Non-Toxic | 0.15 |
| LL1C | 0.002 | 0.00003 | 0.13 | Non-Toxic | 0.00093 | 0.000072 | Non-Toxic | 0.078 | Non-Toxic | Non-Toxic | Non-Toxic | 0.21 |
| LL1D | 0.00085 | 0.000012 | 0.42 | Non-Toxic | 0.00048 | 0.00056 | Non-Toxic | 0.073 | Non-Toxic | Non-Toxic | Non-Toxic | 0.5 |
| LL1E | 0.00053 | 0.0000094 | 0.3 | Non-Toxic | 0.0002 | 0.00011 | Non-Toxic | 0.083 | Non-Toxic | Non-Toxic | Non-Toxic | 0.39 |
| LL1F | 0.051 | 0.00019 | 0.25 | Non-Toxic | 0.00037 | 0.00022 | Non-Toxic | 0.023 | Non-Toxic | Non-Toxic | Non-Toxic | 0.28 |
| LL1G | 0.00041 | 0.000096 | 0.65 | Non-Toxic | 0.00014 | 0.000068 | Non-Toxic | 0.093 | Non-Toxic | Non-Toxic | Non-Toxic | 0.74 |
| LL2A | 0 | 0.0000054 | 0.015 | Non-Toxic | 0.00018 | 0.000045 | Non-Toxic | 0.12 | Non-Toxic | Non-Toxic | Non-Toxic | 0.14 |
| LL2B | 0.0022 | 0.000036 | 0.043 | Non-Toxic | 0.0096 | 0.00049 | Non-Toxic | 0.08 | Non-Toxic | Non-Toxic | Non-Toxic | 0.13 |
| LL2C | 0.000047 | 0.000055 | 0.04 | Non-Toxic | 0.00098 | 0.00025 | Non-Toxic | 0.077 | Non-Toxic | Non-Toxic | Non-Toxic | 0.12 |
| LL2D | 0.00038 | 0.00007 | 0.021 | Non-Toxic | 0.00056 | 0.00053 | Non-Toxic | 0.1 | Non-Toxic | Non-Toxic | Non-Toxic | 0.13 |
| LL2E | 0.000094 | 0.000016 | 0.091 | Non-Toxic | 0.0013 | 0.00018 | Non-Toxic | 0.031 | Non-Toxic | Non-Toxic | Non-Toxic | 0.12 |
| LL2F | 0.00021 | 0.00017 | 0.023 | Non-Toxic | 0 | 0.00033 | Non-Toxic | 0.54 | Non-Toxic | Non-Toxic | Non-Toxic | 0.56 |
| LL2G | 0 | 0.000009 | 0.01 | Non-Toxic | 0 | 0.000048 | Non-Toxic | 0.015 | Non-Toxic | Non-Toxic | Non-Toxic | 0.025 |
| LL3A | 0.0048 | 0 | 0.05 | Non-Toxic | 0.003 | 0.00017 | Non-Toxic | 0 | Non-Toxic | Non-Toxic | Non-Toxic | 0.054 |
| LL3B | 0.0018 | 0.000011 | 0.026 | Non-Toxic | 0.0013 | 0.00031 | Non-Toxic | 0.33 | Non-Toxic | Non-Toxic | Non-Toxic | 0.36 |
| LL3C | 0.00071 | 0 | 0.026 | Non-Toxic | 0.00089 | 0.00022 | Non-Toxic | 0.1 | Non-Toxic | Non-Toxic | Non-Toxic | 0.13 |
| LL3D | 0.0039 | 0.00002 | 0.049 | Non-Toxic | 0.00056 | 0.00036 | Non-Toxic | 0.2 | Non-Toxic | Non-Toxic | Non-Toxic | 0.25 |
| LL3E | 0.0031 | 0.000069 | 0.054 | Non-Toxic | 0 | 0.00047 | Non-Toxic | 0.1 | Non-Toxic | Non-Toxic | Non-Toxic | 0.16 |
| LL3F | 0.0014 | 0.000068 | 0.12 | Non-Toxic | 0 | 0.00067 | Non-Toxic | 0.079 | Non-Toxic | Non-Toxic | Non-Toxic | 0.2 |
| LL3G | 0.0018 | 0.000028 | 0.27 | Non-Toxic | 0.00041 | 0.00031 | Non-Toxic | 0.51 | Non-Toxic | Non-Toxic | Non-Toxic | 0.78 |
